# Novel Compound Heterozygous Mutations in Two Families With Bernard–Soulier Syndrome

**DOI:** 10.3389/fped.2020.589812

**Published:** 2021-01-22

**Authors:** Milen Minkov, Petra Zeitlhofer, Andreas Zoubek, Leo Kager, Simon Panzer, Oskar A. Haas

**Affiliations:** ^1^Department of Pediatrics, Clinic Floridsdorf, Vienna, Austria; ^2^Faculty of Medicine, Sigmund Freud University, Vienna, Austria; ^3^St. Anna Children's Hospital, Department of Pediatric Hematology/Oncology, University Clinic of Pediatrics, Medical University of Vienna, Vienna, Austria; ^4^Labdia GmbH, St. Anna Kinderkrebsforschung GmbH, Vienna, Austria; ^5^Private Practice for General Pediatrics and Pediatric Hematology/Oncology, Maria Enzersdorf, Austria; ^6^Department of Blood Group Serology and Transfusion Medicine, Medical University Vienna, Vienna, Austria

**Keywords:** thrombocytopenia, inherited, platelet disorders, children, Bernard-Soulier syndrome

## Abstract

**Background:** Bernard–Soulier Syndrome (BSS) is a rare autosomal recessive bleeding disorder with large platelets and thrombocytopenia. It is caused by homozygous or compound heterozygous mutations in the *GP1BA, GP1BB*, or *GP9* genes, which together encode the platelet surface receptor glycoprotein complex GPIb-IX-V.

**Objectives:** We report two novel heterozygous mutations in the *GP1BA* and the *GP9* genes, respectively.

**Patients/Methods:** We analyzed the platelet glycoprotein expression by flow cytometry and screened the relevant genes for responsible mutations in two unrelated families.

**Results:** Flow cytometric analyses revealed the absence of CD42a (GPIX) and CD42b (GPIb) on the platelets in the two affected siblings of family 1 and a significantly reduced expression of CD42b (GPIb) in the patient of family 2. In the two siblings, we identified a known frameshift (c.1601_1602delAT) and a novel nonsense mutation (c.1036C>T) in the *GP1BA* gene that abrogated the production of GP1bα. In the other patient, we found a novel missense mutation (c.112T>C) that was co-inherited with a common one (c.182A>G) in the *GP9* gene, respectively. All analyzed heterozygous carriers were asymptomatic and had a normal GPIb-IX-V expression.

**Conclusions:** The two novel *GP1BA* and *GP9* mutations reported herein increment the number of causative genetic defects in BSS.

## Introduction

Bernard–Soulier Syndrome (BSS; OMIM ID #231200), is a rare autosomal recessive inherited bleeding disorder that is characterized by thrombocytopenia and large platelets (1, 2). The bleeding tendency in BSS results from the functional impairment of the glycoprotein complex Ib-IX-V (GPIb-IX-V), which consists of four polypeptide chains (GPIbα, GPIbβ, GPIX, and GPV) that are assembled in a ratio of 2:4:2:1 (3–5). As a receptor for the von Willebrand factor (vWF), this complex is crucial for platelet adhesion and activation. The four polypeptide chains are coded by the genes *GP1BA, GP1BB, GP9*, and *GP5*, respectively.

Homozygous or compound heterozygous mutations in *GP1BA, GP1BB*, or *GP9* genes are responsible for the autosomal recessive forms and mutations in the *GP1BA* gene for the rare autosomal dominant trait (6–9), whereas none are known in the *GP5* gene (10).

Herein, we report three compound heterozygous BSS cases from two families in which we identified two novel mutations in the *GP1BA* and the *GP9* genes, respectively.

### Patients

#### Family 1

**Figure 1:**
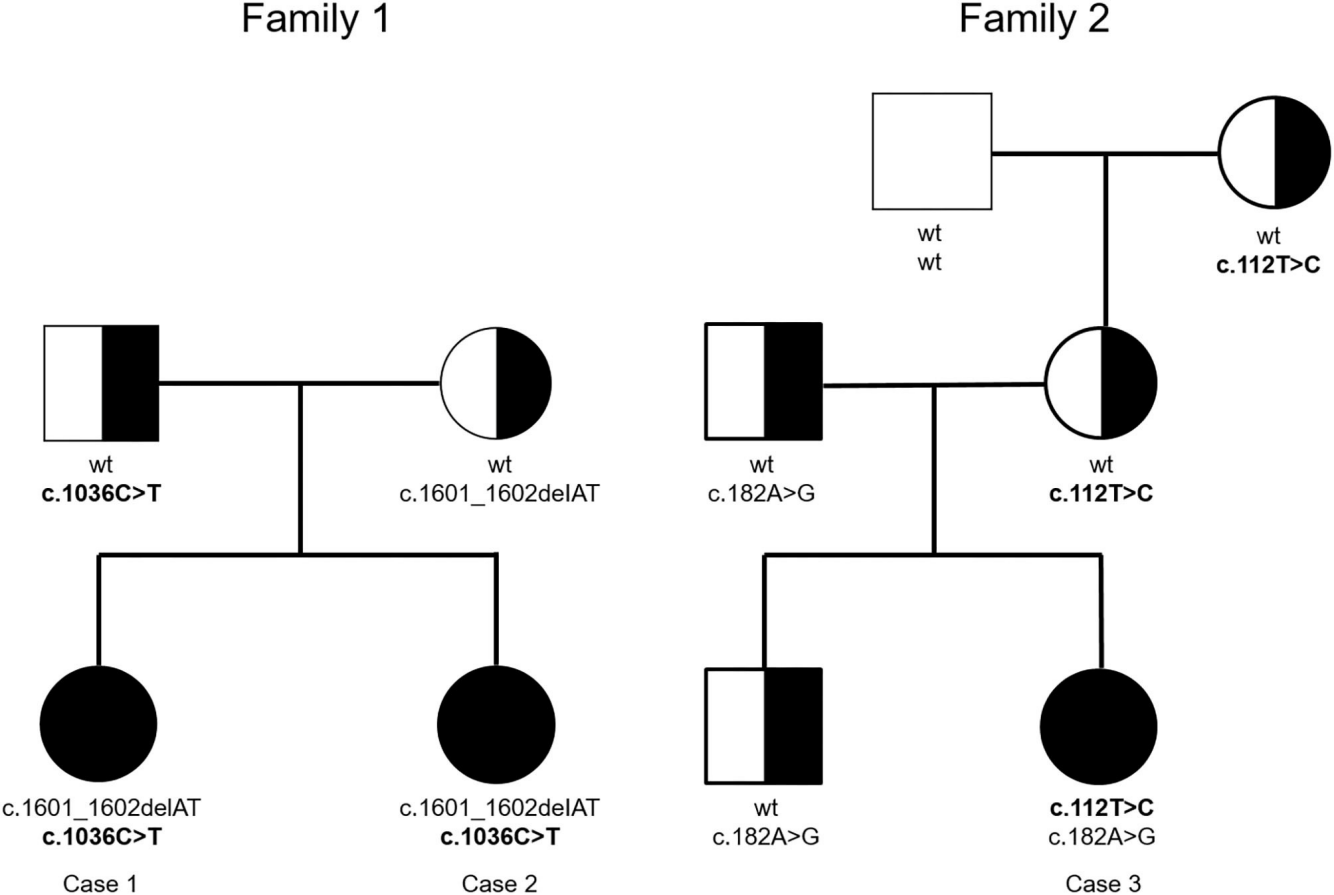
Pedigrees of the two BSS families (novel mutations depicted in bold).

The pedigrees of Family 1 and Family 2 are shown in Figure 1.

##### Case 1

The index patient of this family is a now 11-year-old girl that was born to healthy non-consanguineous parents after an uneventful pregnancy at 40 weeks of gestation with a birth weight of 3.455 kg and a height of 54 cm. A diagnostic workup for febrile illness at the age of 13 months revealed thrombocytopenia with large platelets (61 × 10^9^/L, MPV 17 fl) but no leukocyte inclusion bodies. There was no bleeding history in the family. Nevertheless, based on platelet morphology, inherited thrombocytopenia was suspected and flow cytometry followed by genetic testing were performed. Over the years, the platelet counts remained in the range of 40 to 70 × 10^9^/L. Aside from petechial rashes and recurring mild epistaxis (mostly left-sided due to a prominent plexus Kiesselbach), she experienced three episodes of prolonged bleeding during the first 6 years of age that required medical interventions. Two of these were successfully controlled with local and systemic application of tranexamic acid, whereas the third one, which was due to oral mucosa laceration by potato chips, required transfusion of platelets and packed red blood cells for a particularly dramatic severe blood loss.

##### Case 2

The sister of the index case, now 8 years old, presented with bruises and thrombocytopenia already at birth. The positive family history for BSS allowed for a straightforward workup. The platelet counts remained between 40 and 70 × 10^9^/L. She also had intermittent petechial rashes predominantly on the face and the upper extremities as well as two episodes of prolonged mucosal bleeding that also required medical interventions. At the age of four, she had a respiratory infection with bilateral epistaxis, which was originally treated locally with cooling, decongestants and oral tranexamic acid but then, prolonged bleeding and a drop of the hemoglobin level to 8.3 g/dl required hospitalization for nasal tamponade as well as transfusion of platelets and packed red blood cells. 6 months later, she was again admitted to the hospital because of continuous bleeding after an incidental injury of the lip and gum, which stopped after intravenous tranexamic acid application and a platelet transfusion.

#### Family 2

##### Case 3

The index patient of the second family was the first child of non-consanguineous healthy parents. The girl was born after an uneventful pregnancy at week 35 of gestation with a birth weight of 2.245 kg and a height of 45 cm. She was admitted to a neonatal intensive care unit because of a non-severe transitory asphyxia (Apgar 3/5/10), where thrombocytopenia with a platelet count of 51 × 10^9^/L was noted. There were no obvious signs of external bleedings and, in particular, also no intracranial one. There was no bleeding history in the family. The tests for intrauterine infections were negative. Both parents had normal platelet counts (237 and 204 × 10^9^/L, respectively). Neonatal alloimmune thrombocytopenia was excluded by negative MAIPA test of the maternal serum (11) and negative cross-match with paternal platelets. An X-ray examination of both forearms excluded radius anomaly. Since her platelet count dropped to 30 × 10^9^/L, she received one unit of platelets and four infusions of intravenous immunoglobulin (400 mg/kg/day for 4 days) at the neonatology unit, which increased the platelet count to 90 × 10^9^/L. During her first year of life the platelet counts remained between 50 and 120 × 10^9^/L. At the age of 14 months, she was referred to our department for further diagnostic evaluation of the persistent thrombocytopenia.

At admission, her platelet count was 60 × 10^9^/L, the MPV 12 fl. Morphological assessment of the blood smear showed a considerable proportion of large platelets without neutrophilic inclusion bodies. The proportion of reticulated platelets measured by flow cytometry was 29.1% (normal range 2.0–7.0%). She had no platelet-reactive antibodies and the number and morphology of mature megakaryocytes in her bone marrow was normal. In addition, coagulation tests (prothrombin time, aPTT, thrombin time, fibrinogen, vWF antigen, and vW-Ristocetin co-factor activity) were normal. Platelet function tests were performed to exclude von Willebrand disease Type 2B. Platelet aggregation in response to ristocetin 1.2 mg/ml and 0.9 mg/ml was 30 and 12%, respectively. The aggregation response to the other agonists was between 61 and 84%. Her maternal grandfather had mantle cell lymphoma but otherwise the family history was inconspicuous. Although the combination of thrombocytopenia with large platelets suggested an underlying genetic condition, further clarification deemed unnecessary at that time, as platelet counts remained stable without clinical bleeding signs.

Her brother, who was born 2 years later, experienced also a transient thrombocytopenia at the age of 2 months, which resolved spontaneously within a few weeks. A laboratory checkup of the boy for a scheduled adenotomy at the age of 5 years revealed a prolonged activated partial thrombin time (aPTT). Hemophilia and von Willebrand disease were excluded and the aPTT normalized spontaneously. Based on this episode, the parents requested to investigate the cause of their first-born child's thrombocytopenia and, in particular, its potential relationship with her brother's problem.

We therefore screened the respective BSS genes for causative mutations in the index patient, who in the meantime is 21 years old, her brother, both parents, as well as her maternal grandparents. Except for prolonged menstrual bleedings that are well controlled by oral contraceptives, she had not endured any significant bleedings so far.

## Methods

### Platelet Morphology and Platelet Function Tests

Platelet and white blood cell morphology were assessed on May-Grünwald-Giemsa stained peripheral blood smears in all patients.

Platelet function tests were performed in Case 3 for a suspected von Willebrand disease Type 2B. Agonists' inducible platelet aggregation in platelet-rich plasma was studied with a Platelet Aggregation Profiler 8E (Bio/Data Corporation, Horsham, PA). Agonists (möLab Langenfeld, Germany) were ADP 10 μM and 5 μM, arachidonic acid 1.6 mM, collagen 190 μg/ml, and ristocetin 1.2 mg/ml and 0.9 mg/ml. Platelet aggregation was monitored for 10 min, and maximal aggregation was recorded.

### Flow Cytometry

All flow cytometry evaluations were performed on Trisodium citrate-anticoagulated whole blood (9 parts of whole blood, 1 part of 0.108 mol/L trisodium citrate) with a FACSCalibur flow cytometer (Becton Dickinson, BD, San Jose, CA) and an argon laser at 488 nm (12). In brief, GP surface expression was examined by staining 20-μl aliquots of diluted blood samples with the following monoclonal antibodies (MoAb): Fluorescein (FITC)-labeled anti-CD42a (clone ALMA.16, BD), phycoerythrin (PE)-labeled anti-CD42b (clone SZ2, Immunotech, Marseille, France), allophycocyanin (APC)-labeled anti-CD41a (clone HIP8, BD), FITC-labeled anti-CD61 (Immunotech), and FITC-labeled anti-CD36 (clone FA6.152, Immunotech) for 15 min. The reaction was stopped by adding 500 μl of ice-cold phosphate-buffered saline. Standard beads that contain specific amounts of “mean equivalent soluble fluorescein molecules” were used for calibration. A total of 10,000 events were acquired in a logarithmic mode within the platelet window. For analyses, we gated on SSC vs. fluorescence 4 (FL4). Specific binding of the MoAb was calculated by subtracting non-specific binding of isotype-matched controls. The results (Table 1) are expressed as median fluorescence intensity (MFI). Samples from two healthy controls were analyzed concurrently.

**Table 1:** Platelets' glycoprotein expression of flow cytometry^*^.

**Studied markers**	**Family 1**				**Family 2**				**Controls**
	**Case 1**	**Case 2**	**Mother**	**Father**	**Case 3**	**Sibling**	**Mother**	**Father**	*n* = 13^**^
CD42a (GPIX)	**131**	**88**	939	1055	**67**	626	736	491	1149 (755–1556)
CD42b (GPIbα)	**9.0**	**7.0**	162	189	**55**	429	466	422	374 (281–486)
CD41 (GPIIb, integrin αIIb)	673	1263	552	537	965	425	610	500	465 (427–574)
CD61 (GPIIIa, integrin β3)	615	649	532	567	186	72	84	62	437 (156–508)
CD36 (GPIV, thrombospondin receptor)	505	482	237	392	291	246	276	179	234 (124–306)

* Data are given as median fluorescence intensity (MFI);

** *data from healthy adult controls are provided as median and range (in brackets). Bold numbers indicate low MFI values*.

### Molecular Genetic Analyses

For genetic testing, we obtained the informed consent from all adults and the children's parents. Genomic DNA was isolated from EDTA blood samples using the NucleoSpin® Blood kit from Macherey-Nagel (Düren, Germany). The coding regions of the *GP1BA* (index patients of both families) and *GP1BB* and *GP9* (index patient of family 2) genes were amplified by PCR and the purified DNA fragments were subsequently Sanger sequenced. The primer sequences are available upon request. To determine the carrier status of all other family members, we only looked for the mutations that had been identified in the respective index patients.

The reference sequence of the *GP1BA* gene is NM_000173.5, that of the *GP1BB* gene is NM_000407.4, and that of the *GP9* gene is NM_000174.4, with nt +1 being the A of the initiation codon. Allele frequencies were extracted from the gnomAD browser (genome Aggregation Database, v2.1.1; http://gnomad.broadinstitute.org; accessed July 21, 2020). Where applicable, computational algorithms used to assess the potential effect of the variant were PolyPhen2 (13), SIFT (14), Mutation Taster (15), and CADD (16).

## Results

### Platelet Morphology

**Figure 2:**
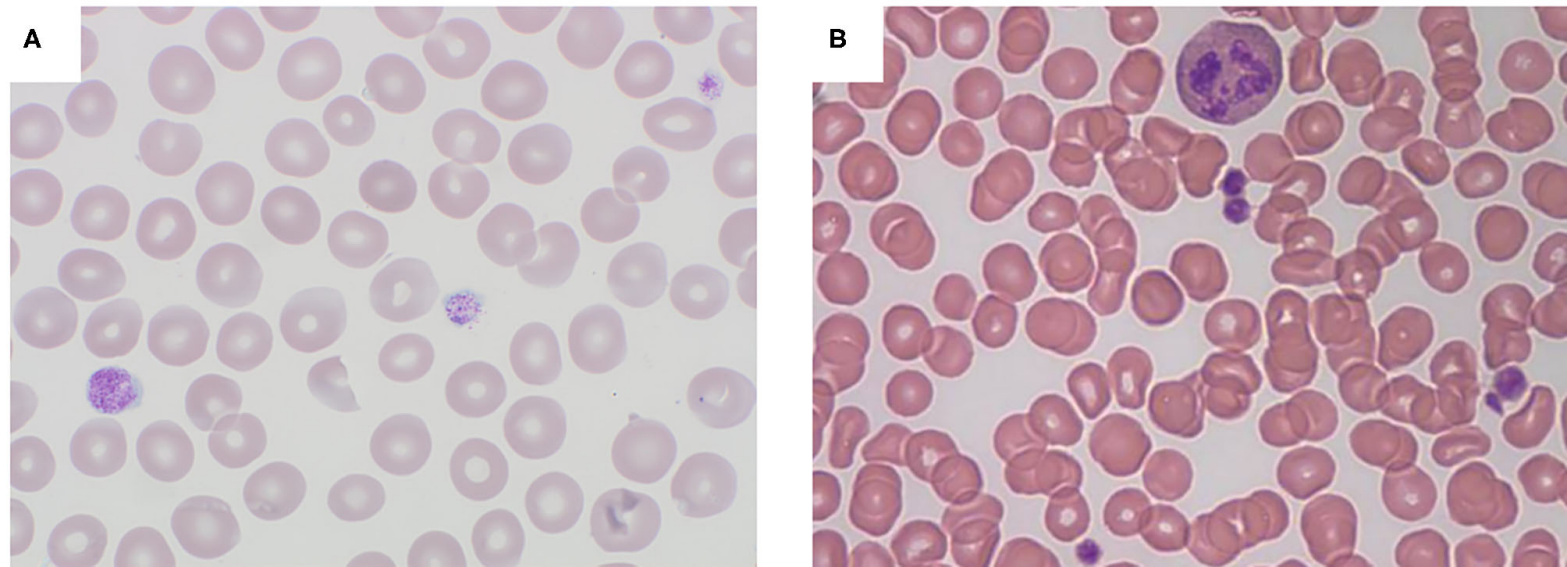
Peripheral blood smears (magnification × 1000): **(A)** Case 1 illustrating giant platelets; **(B)** Case 3 demonstrating less prominent large-sized platelets.

The blood films of all three patients revealed platelets obviously larger than normal, but platelet size in case 3 was less prominent and could be easily overlooked by an inexperienced observer (Figure 2).

### Flow Cytometry

**Figure 3:**
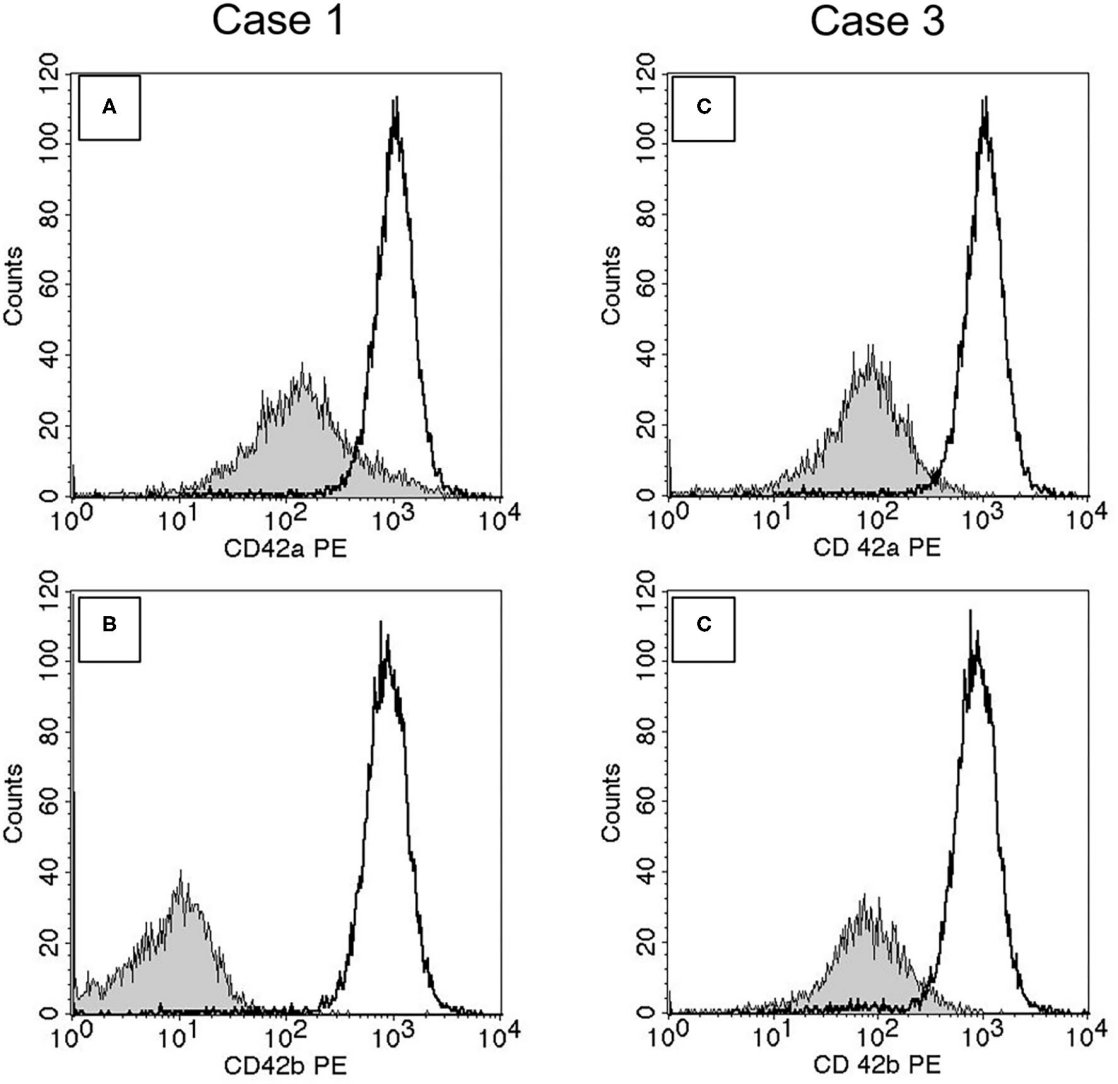
Flow cytometry of the index cases of the two families: Plots **(A)** CD42a and **(B)** CD42b in Case 1; **(C)** CD42a and **(D)** CD42b in Case 3. The histograms of the cases and the same control are overlayed. The shaded area represents the histogram of the index case, the open area of the control.

The results of both families are summarized in Table 1. Consistent with a diagnosis of BSS, the expressions of CD42a (GPIX) and CD42b (GPIb) were significantly reduced in the compound heterozygous patients from both families, compared to the heterozygous relatives and the controls. The plots of the index cases (Case 1 and Case 3) are presented in Figure 3. Of interest, the expression of the other glycoproteins in the patients was higher than in the heterozygous carriers, who had normal expression of the respective platelet surface glycoproteins.

### Molecular Genetics

#### Family 1 (Case 1 and Case 2)

The coding region of the *GP1BA* gene was sequenced in Case 1. Compound heterozygosity for a known pathogenic 2-base deletion c.1601_1602delAT (p.Tyr534CysfsTer82) (17) and a novel c.1036C>T nonsense mutation that created a stop codon (p.Gln346Ter) was found (Supplementary Figure 1a). This particular variant is obviously very rare, as it has not been reported in gnomAD. Genetic testing of her sister (Case 2) confirmed that she also has the same compound heterozygous mutations, whereas the mother carries the 2-base deletion and the father, the novel nonsense mutation (Figure 1).

#### Family 2 (Case 3)

Since sequencing of the coding region of the *GP1BA* and the *GP1BB* genes in the patient of family 2 did not uncover any mutations, we also analyzed the coding exon 3 of the *GP9* gene and identified two compound heterozygous missense mutations, an already known one, c.182A>G (p.Asn61Ser), and a novel one, c.112T>C (p.Cys38Arg) (Supplementary Figure 1b). The c.182A>G mutation is the most commonly encountered one in the *GP9* gene (18), whereas the c.112T>C mutation, which is predicted to be damaging by computational algorithms, has not yet been described and is not listed in gnomAD. The carrier screening of the other family members revealed that her brother and father have the common mutation and her mother and maternal grandmother have the novel one (Figure 1). The results of the molecular tests are summarized in Table 2.

**Table 2:** Results of the molecular genetic testing.

**GENE^a^**	**POS^b^**	**Transcript^c^**	**AA^d^**	**EFFECT^e^**	**SNP ID^f^**	**gnomAD MAF^g^**	**PolyPhen2^h^**	**SIFT^i^**	**MutationTaster^j^**	**CADD^k^**
*GP1BA*	17:4836935	**NM_000173.5:c.1036C>T**	p.Gly346Ter	Stop gain	-	-	NA	NA	Damaging	32.0
*GP1BA*	17:4837500–4837501	NM_000173.5:c.1601_1602delAT	p.Tyr534CysfsTer82	Frameshift	-	-	NA	NA	Damaging	29.5
*GP9*	3:128780694	**NM_000174.4:c.112T>C**	p.Cys38Arg	Missense	rs764292260	-	Probably damaging	Damaging	Damaging	24.4
*GP9*	3:128780764	NM_000174.4:c.182A>G	p.Asn61Ser	Missense	rs5030764	0.00051	Probably damaging	Damaging	Damaging	23.4

a *The identified gene(s) symbol (GENE) according to the HUGO Gene Nomenclature Committee (https://www.genenames.org/)*.

b *The position (POS) of the identified variant(s) in the GRCh37/hg19 assembly (https://www.ncbi.nlm.nih.gov/assembly/GCF_000001405.13/)*.

c *Alteration on the transcript (TRANSCRIPT) for which the gDNA and protein change are described*.

d *The amino acid change (AA) annotated following the guidelines from the Human Genome Variation Society (http://varnomen.hgvs.org/)*.

e *The effect (EFFECT) that is caused by the variant given by the snpeff annotation in the transcript (http://snpeff.sourceforge.net/)*.

f *The primary identifier for variants (SNP_ID) in the Single Nucleotide Polymorphism Database (dbSNP) of Nucleotide Sequence Variation (https://www.ncbi.nlm.nih.gov/books/NBK21088/)*.

g *The minor allele frequency (gnomAD MAF) provided for each specific variant in all populations/ethnicities in the genome Aggregation Database v2.1.1 (http://gnomad.broadinstitute.org; accessed 21 July 2020)*.

h *Prediction program: PolyPhen2 (http://genetics.bwh.harvard.edu/pph2/) (13)*.

i *Prediction program: SIFT (http://sift.jcvi.org/) (14)*.

j *Prediction program: MutationTaster (http://sift.jcvi.org/) (15)*.

k *Prediction program: CADD (http://cadd.gs.washington.edu/) (16)*.

*NA not applicable; novel mutations are depicted in bold*.

## Discussion

The triad thrombocytopenia and large platelets together with a reduced or absent expression of components of the GPIb-IX-V complex is an unambiguous diagnostic hallmark of BSS, an inborn bleeding disorder that can be caused by mutations in three different genes and be inherited in either a rare autosomal dominant or the more common autosomal recessive manner (6–8, 19). Given the genetic heterogeneity, it is therefore of utmost importance to identify the specific underlying genetic defect, not only to establish an indisputable diagnosis, but especially also to obtain the information that is essential for a precise carrier identification and a trustworthy counseling of family members. Based on these necessities, we genetically tested three patients with a clinical diagnosis of BSS and identified two novel mutations, one in the *GP1BA* and another one in the *GP9* gene, each of which occurred together with previously known ones.

The “Leiden Open Variation Database (LOVD)” currently lists 558 individuals with BSS, 366 (66%) of which result from mutations in the *GP1BA*, 73 (13%) in the *GP1BB*, and 119 (21%) in the *GP9* gene (Supplementary Table 1). They are scattered over the entire genes without any evident hotspots and comprise missense, nonsense as well as frameshift mutations (19). Since many BSS cases occur in consanguineous families, approximately 85% of them are homozygotes and only approximately 13% are compound heterozygotes (19). Although individuals with only one mutant allele are overwhelmingly asymptomatic carriers, approximately 2% of them, primarily those with a *GP1BA* mutation, can have a mild macrothrombocytopenia that is transmitted as an autosomal dominant trait (19). The majority of these monoallelic dominant cases share the same c.515C>T (Bolzano) founder mutation, which is particularly prevalent in Italy (9).

The two compound heterozygote siblings of family 1 had a novel nonsense c.1036C>T mutation in the *GP1BA* gene, which produces a stop codon. It occurred together with a previously known 2-base deletion (c.1601_1602delAT) causing the frameshift mutation p.Tyr534CysfsTer82 (17). The *GP1BA* gene encodes GPIbα, a type I transmembrane protein, which binds to two GPIbβ subunits and forms the GPIb complex that, in turn, interacts non-covalently with the GPIX and GPV subunits (20). Functional studies have already shown that the subunits GPIb and GPIX are both required for an efficient surface expression of the GPIb-IX-V complex (20, 21).

As evident from the flow cytometry data in our patients, these mutations lead to an almost complete loss of GPIb expression on the platelet surface. The frameshift mutation is particularly common in patients of northern European ancestry (17, 22). It causes a truncated GPIbα with modified transmembrane and cytoplasmic domains. While this truncated version is lacking on platelets, it is nevertheless present in the plasma of patients, which indicates that the mutant protein may either be poorly anchored or that it is proteolytically cleaved (17). Although we have not studied the functional consequences of the novel mutation *in vitro* or in an animal model, the predicted truncation of the protein after translation of 345 amino acids clearly indicates that both the transmembrane and the cytoplasmic domains of the mutated GPIb are lost.

Both missense mutations that we found in the index patient of family 2 in exon 3 of the *GP9* gene affect the protein sequence of the extra-cytoplasmic domain between the N-terminus and the leucine-rich repeats of the GP IX polypeptide. The paternal-derived mutation c.182A>G is not only the most common one in GP9 (23), but it is also an ancient one that is scattered throughout Northern and Central Europe (24). It replaces asparagine with serine, which affects the buried leucine-rich repeat consensus residue and thus protein folding. The novel maternal-derived mutation c.112T>C, on the other hand, replaces a cysteine that is involved in a conserved disulfide bond by arginine. Based on the criteria provided on the Varsome site (https://varsome.com/variant/hg19/rs764292260), we classified this sequence variant as “likely pathogenic.” We hypothesize that, in analogy with the effects of such *GP1BB* mutations, the two *GP9* missense mutations obstruct the proper folding of the protein, which then impede the formation of a functional GPIX and subsequently cause an incorrect assembly of the GPIb-IX complex (25–28). The flow cytometry findings in case 3 suggest that the GPIX misfolding results in partial lysosomal degradation of GPIbα, as previously shown by Dong et al. (20).

Genotype/phenotype studies in compound heterozygous patients are still lacking (29). However, given the data available even in homozygous BSS patients, a clear correlation between genetic defects and clinical severity of compound heterozygous BSS is unlikely to be achieved. The two patients of family 1 who lacked GPIb expression on platelets had continuously mild spontaneous bruises as well as mucosal bleedings that required medical interventions. The patient from family 2, on the other hand, experienced apart from prolonged menstrual bleeding no other clinically relevant bleedings. In this instance, one may therefore speculate that the milder clinical course may possibly be ascribed to a partially retained GPIb-IX-V function.

## Data Availability Statement

The GP1BA and GP9 mutations described in this report have been deposited in the Leiden Open Variation Database (LOVD) at http://www.lovd.nl/GP1BA and http://www.lovd.nl/GP9 in January 2019.

## Ethics Statement

Ethical review and approval was not required for the study on human participants in accordance with the local legislation and institutional requirements. Written informed consent to participate in this study was provided by the participants' legal guardian/next of kin.

## Author Contributions

MM: drafted the manuscript and was actively involved in the diagnostic workup, providing clinical care of the patients, and counseling the families. PZ: wrote the section methods with respect to genetic testing, performed and interpreted the genetic tests, and searched and analyzed available genetic databases. AZ: provided outpatient care, delivered follow-up information on family 1, and contributed to the case report section. LK: provided outpatient care, delivered follow-up information on family 2, and contributed to the case report section. SP: performed platelet flow cytometry and platelet aggregation tests, wrote the respective part of methods, interpreted its results, and contributed to the introduction and discussion sections. OH: supervised genetic testing, interpreted the respective results, and contributed to all manuscript sections, with particularly substantial contribution to the discussion section. All authors read critically the manuscript draft, provided input in their area of expertise, and approved the final manuscript version.

## Conflict of Interest

PZ and OH, who are employed in Labdia GmbH, which is a 100% non-profit subsidiary of the Children's Cancer Research Institute (St. Anna Kinderkrebsforschung GmbH), have no commercial or financial relationships that could be construed as a potential conflict of interest. The remaining authors declare that the research was conducted in the absence of any commercial or financial relationships that could be construed as a potential conflict of interest.

## Supplementary Material

Supplementary Figure 1 Mutations in the *GP1BA* and *GP9* genes: **(a)** *GP1BA* electropherograms of the wild-type (upper panel) and the compound heterozygous novel c.1036C>T and c.1601_1602delAT mutations, respectively, that were identified in the index patient of family 1 (lower panel). The sequence of each genotype with the corresponding amino acid residues is shown below. Her also affected sister had the same mutations (data not shown). **(b)** *GP9* electropherograms of the wild-type (upper panel) and the compound heterozygous novel c.112C>T and common c.182A>G mutations (lower panel), respectively, of the affected patient from family 2.
