# Supplementary figures and images for: Novel Compound Heterozygous Mutations in Two Families With Bernard–Soulier Syndrome
Source: Front Pediatr. 2021 Jan 22;8:589812. doi: 10.3389/fped.2020.589812 (PMC7864212; doi:10.3389/fped.2020.589812)

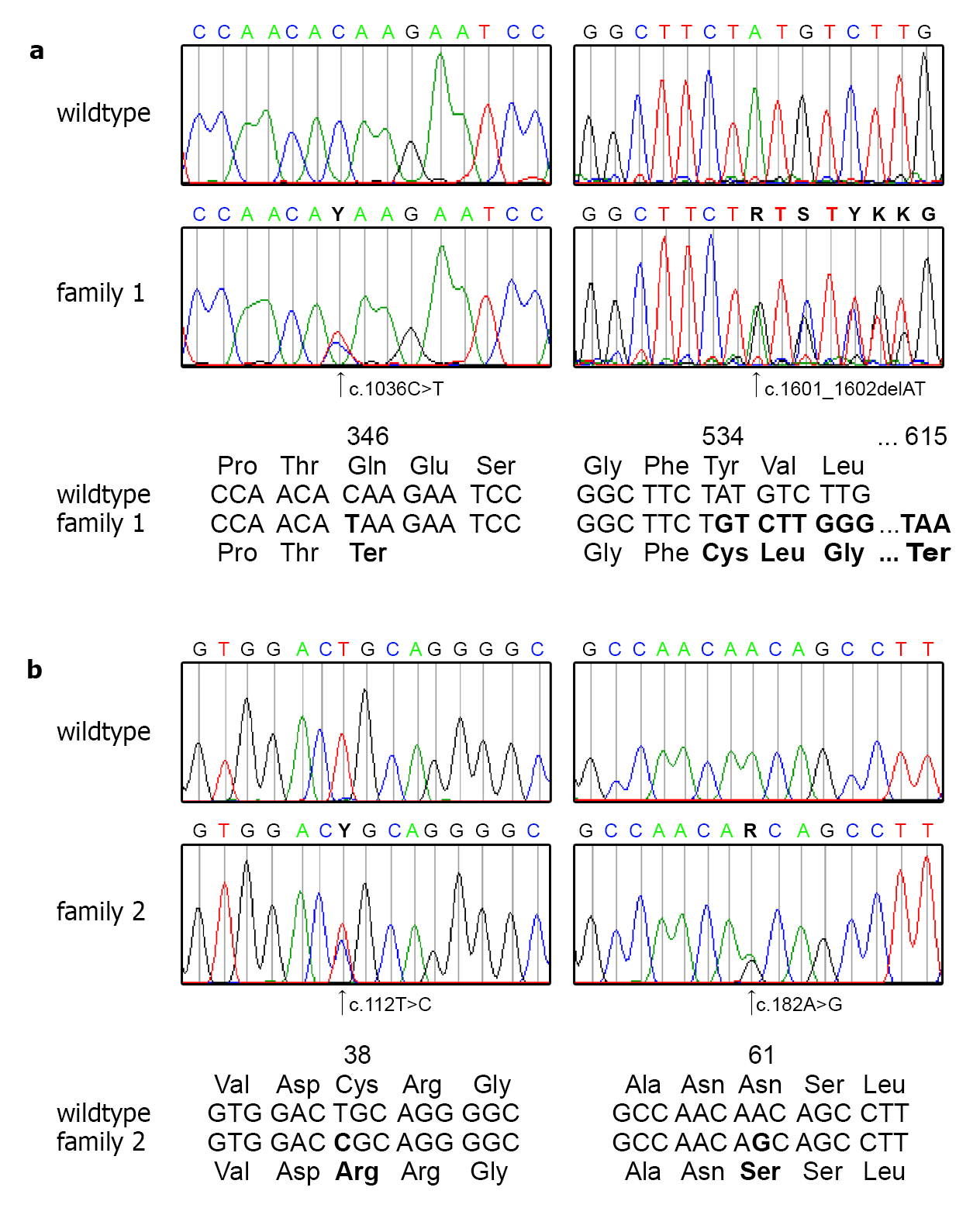

Supplement: Supplementary Figure 1 — Mutations in the GP1BA and GP9 genes: (a) GP1BA electropherograms of the wild-type (upper panel) and the compound heterozygous novel c.1036C>T and c.1601_1602delAT mutations, respectively, that were identified in the index patient of family 1 (lower panel). The sequence of each genotype with the corresponding amino acid residues is shown below. Her also affected sister had the same mutations (data not shown). (b) GP9 electropherograms of the wild-type (upper panel) and the compound heterozygous novel c.112C>T and common c.182A>G mutations (lower panel), respectively, of the affected patient from family 2. [file Image_1.TIF]
