# Supplementary material for: Novel Compound Heterozygous Mutations in Two Families With Bernard–Soulier Syndrome
Source: Front Pediatr. 2021 Jan 22;8:589812. doi: 10.3389/fped.2020.589812 (PMC7864212; doi:10.3389/fped.2020.589812)
Supplement: Supplementary file 2 [file Table_1.DOCX]

**Supplemental Table 1.** Gene-specific frequency and allocation of pathogenic variants in 558 Bernard-Soulier syndrome cases*

|  | **GP1BA** | **GP1BB** | **GP9** |
| --- | --- | --- | --- |
| Individuals with public variants | 366 (66%) | 73 (13%) | 119 (21%) |
| Total number of public variants reported | 154 | 80 | 105 |
| Unique public variants reported | 78 | 46 | 28 |
| All pathogenic variants | 98 | 76 | 103 |
| Unique pathogenic variants | 54 | 42 | 28 |
| All pathogenic recessive variants | 65 | 73 | 99 |
| Unique pathogenic recessive variants | 41 | 40 | 28 |
| All pathogenic dominant variants | 19 | - | - |
| Unique pathogenic dominant variants | 9 | - | - |

*Information extracted from the following databases: lovd.nl/shared/genes/GP1BA, lovd.nl/shared/genes/GP1BA and lovd.nl/shared/genes/GP9 (last accessed on July 17, 2020)
